# Detection of Nonverbal Synchronization through Phase Difference in Human Communication

**DOI:** 10.1371/journal.pone.0133881

**Published:** 2015-07-24

**Authors:** Jinhwan Kwon, Ken-ichiro Ogawa, Eisuke Ono, Yoshihiro Miyake

**Affiliations:** Department of Computational Intelligence and Systems Science, Tokyo Institute of Technology, Yokohama, Kanagawa, Japan; University of Portsmouth, UNITED KINGDOM

## Abstract

Nonverbal communication is an important factor in human communication, and body movement synchronization in particular is an important part of nonverbal communication. Some researchers have analyzed body movement synchronization by focusing on changes in the amplitude of body movements. However, the definition of “body movement synchronization” is still unclear. From a theoretical viewpoint, phase difference is the most important factor in synchronization analysis. Therefore, there is a need to measure the synchronization of body movements using phase difference. The purpose of this study was to provide a quantitative definition of the phase difference distribution for detecting body movement synchronization in human communication. The phase difference distribution was characterized using four statistical measurements: density, mean phase difference, standard deviation (SD) and kurtosis. To confirm the effectiveness of our definition, we applied it to human communication in which the roles of speaker and listener were defined. Specifically, we examined the difference in the phase difference distribution between two different communication situations: face-to-face communication with visual interaction and remote communication with unidirectional visual perception. Participant pairs performed a task supposing lecture in the face-to-face communication condition and in the remote communication condition via television. Throughout the lecture task, we extracted a set of phase differences from the time-series data of the acceleration norm of head nodding motions between two participants. Statistical analyses of the phase difference distribution revealed the characteristics of head nodding synchronization. Although the mean phase differences in synchronized head nods did not differ significantly between the conditions, there were significant differences in the densities, the SDs and the kurtoses of the phase difference distributions of synchronized head nods. These results show the difference in nonverbal synchronization between different communication types. Our study indicates that the phase difference distribution is useful in detecting nonverbal synchronization in various human communication situations.

## Introduction

Human communication consists of verbal and nonverbal communication. In particular, nonverbal communication contributes to human communication in a variety of ways and is an important factor in social interaction [1, 2, 3]. Nonverbal responses in human communication are known to be immediate and honest [4]. Among nonverbal behaviors that contribute to human communication, nodding the head plays an important role as a form of feedback in human communication [5–7].

Of specific relevance to this study, body movement synchrony is known to be a dominant characteristic in nonverbal communication. In face-to-face communication, the synchronization of body movements has been observed in social and interpersonal relationships. For example, the body movements of neonates synchronize with the speech of their mothers [8], and intimate partners frequently interact by synchronizing their postures and body movements [9]. In particular, Hove and Risen (2009) concluded that interpersonal synchrony is the critical factor contributing to likability with an increase of affiliation [10] and Marsh et al. (2009) have reported that behavioral and embodied methods can be used for investigating the relationship between sociality and coordination with other individuals, which is fundamental and serves as the basis for our social connection to others [11]. In addition, the strong synchronization of body movements between clients and their psychotherapy counselors has been found for positively evaluated counseling groups [12]. Specifically, head nods often occur at the same time, even during conversations among multiple participants [6]. Some researchers report that the synchronization of head nods reflects positive emotions in interpersonal relationships [6, 7].

Previous researchers have used video-based analysis to measure body movement synchronization [6–9, 12–17]. Bernieri (1988a) and Bernieri et al. (1988b) have analyzed body movement synchrony by observer ratings through movement synchrony perceived in video clips [16, 17]. In addition, some researchers have analyzed the synchronization of body movements by focusing on changes in the amplitude of body movements with a predetermined video frame rate [12–15]. However, there is no research on phase difference as an indicator of body movement synchronization in human communication. In theoretical studies, phase difference is a very important factor in synchronization analysis because it shows the most accurate temporal relationships in synchronization [18–20]. Therefore, it is necessary to measure phase differences in the synchronization of body movements and to define nonverbal synchronization quantitatively according to the distribution of phase differences.

The purpose of this study was to provide a new method using the phase difference distribution for detecting body movement synchronization in human communication. We characterized the synchronization of body movements using four statistical measurements of the phase difference distribution. These four measurements include: density as an indicator of the synchronization activity, mean phase difference as an indicator of the synchronization direction, and standard deviation (SD) and kurtosis as indicators of the synchronization strength.

To confirm the validity of our definition, we applied our method to human communication in which the roles of speaker and listener were defined. Specifically, we focused on differences in body movement synchronization under different types of communication situations: direct face-to-face communication and remote communication via television. From previous studies, body movements are coordinated between perceptually coupled individuals [20]. Furthermore, Bernieri (1988a) and Bernieri et al. (1988b) have emphasized the importance of interpersonal interaction in body movement synchrony by comparing genuine synchrony with true interaction and pseudo-synchrony with no interaction [16, 17]. Therefore, in this study, we examined the difference in phase difference distribution between face-to-face communication with visual interaction and remote communication with unidirectional visual perception. The face-to-face communication condition is set up as a situation in which two participants are visually coupled, whereas the remote communication condition is set up as a situation in which two participants are not visually coordinated in which the listener has visual information about the speaker but the speaker has no visual access to the listener.

In the materials and methods section, we describe the two types of communication conditions and define the method of detecting phase differences over the whole communication period, as well as the analysis of synchronization using the phase difference distribution, which is characterized through four statistical measurements. In the results section, we describe whether differences in the synchronization between the two types of communication situations were found through the four statistical measurements. In the discussion section, we discuss the effectiveness of our defined method for detecting synchronization.

## Materials and Methods

### Experimental Designs

We used a lecture task in this study to distinguish clearly between the speaker and listener during the communication process. By having the participants perform the lecture task twice in the face-to-face communication and remote communication conditions, we allowed the listener to adapt to the task and to predict the content of the task by the learning effect. Therefore, pairs of participants were divided into two groups, and they performed the lecture task separately. In the face-to-face communication condition, a teacher who takes over the role of speaker delivered certain content to a student who takes over the role of listener in face-to-face communication. Then, we used head nodding motion as the specific indicator to clarify the mechanism of embodied synchrony in human communication and attached an acceleration sensor with high temporal resolution directly to participants’ forehead to analyze their body movement details. We extracted a set of phase differences from the time-series data on the acceleration of head nods between two participants throughout the lecture task and we detected the synchronization of head nods from the distribution of the phase differences. In the remote communication condition, a pair of participants performed the lecture task remotely (in different rooms) via television and again we detected the synchronization of head nods from the phase difference distribution in time-series data on the acceleration of head nods between pairs of participants. The size of the teacher’s face, the volume of the teacher’s voice, and the gaze point between the teacher and student were identical in the face-to-face communication and remote communication conditions. The listener was only allowed back-channel signals during the lecture task in the face-to-face communication and remote communication conditions, and the constraints for the lecture task were the same in both experiments.

### Participants

Twelve pairs of subjects (16 males and eight females, all in their 20s) participated in the face-to-face communication and remote communication conditions, respectively. We derived the following selection criteria for pairs of participants from a previous study [7, 21]: the partners should differ in age by less than five years, be of the same sex, and be native speakers of Japanese. In addition, we imposed the condition that only two people would interact with each other during the experiment. The ethics committee of the Tokyo Institute of Technology specifically approved this study, and written informed consent was obtained from each participant to participate in this study.

### Apparatus

**Fig 1:**
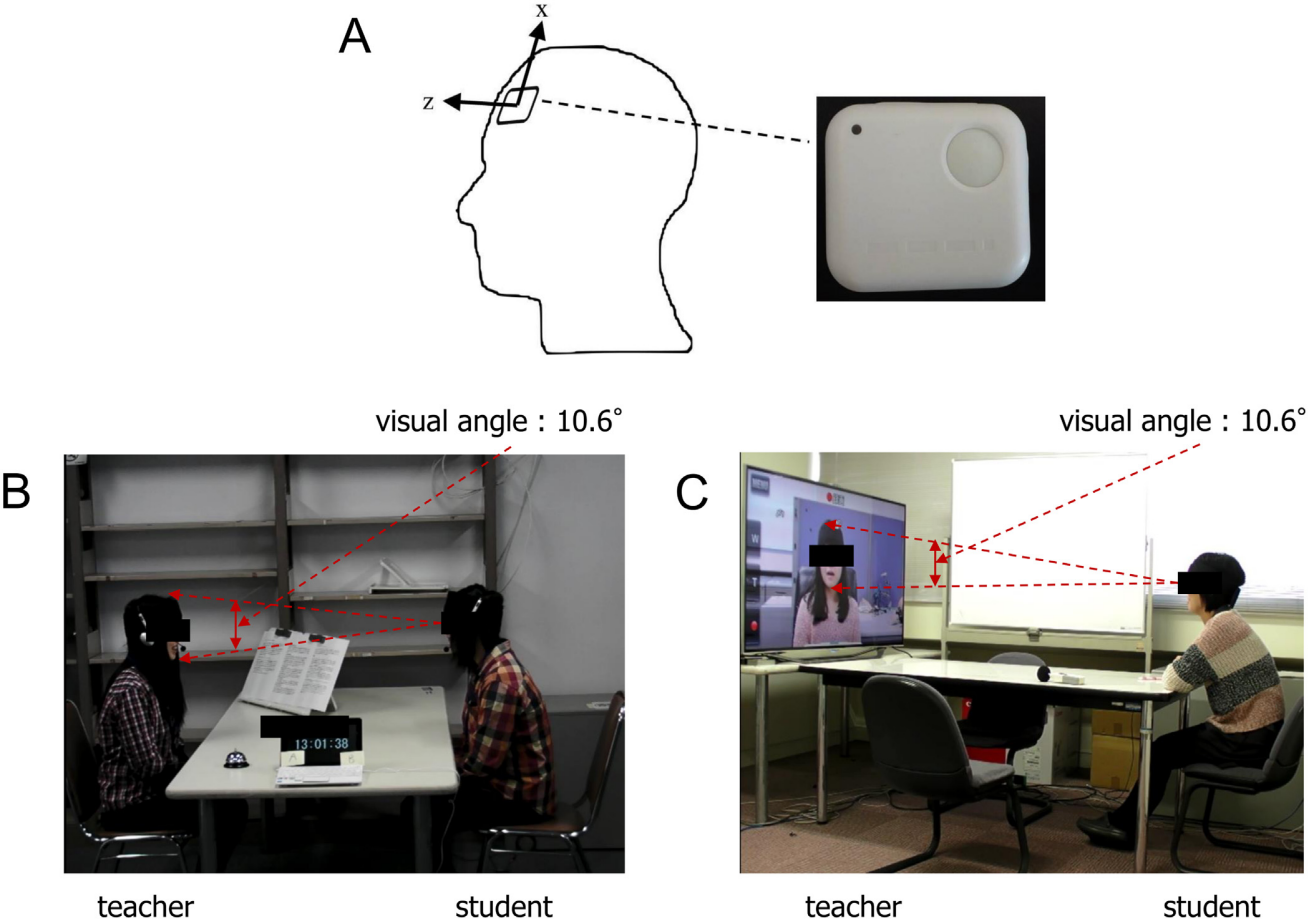
Schematic illustration of the face-to-face communication and remote communication conditions. (A) shows the position of a small three-axis acceleration sensor. The sensor (4.5 cm × 4.0 cm) had a sampling frequency of 100 Hz, and it was attached to the forehead of each participant. Head movement was defined as a movement in the vertical and longitudinal direction. (B) shows the experimental situation in the face-to-face communication condition, and (C) shows the experimental situation in the remote communication condition.

We used a small three-axis acceleration sensor (4.5 cm × 4.0 cm) with a sampling frequency of 100 Hz (WAA-006, Wireless Technologies, Japan) to measure time-series data on the acceleration of head nods. The data were recorded on a PC (Latitude E5400, Dell, TX, USA) via Bluetooth. The acceleration sensor was attached to the forehead of each participant (see Fig 1A). In addition, we used three video cameras (Xacti, Sanyo, Japan) to record the overall situation of the teacher and student participants. In the remote communication condition, a video camera (HDR-CX270, Sony, Japan) in the teacher’s room recorded images of the teacher and transmitted them to a television (60-inch LED display, with 1920 × 1080 pixel resolution, UN60ES8000F, Samsung, Korea) in the student’s room. The video camera and television were connected by an HDMI cable, and another camera (Xacti, SANYO, Japan) recorded the student.

### Experimental Procedures

In the face-to-face communication condition, each participant was randomly assigned to the role of either teacher or student, and before the experiment began, the teacher was given a Wikipedia article. The article was a “cold reading,” related to the techniques of persuasion [22]. The criteria for selecting the article were that it should be on a less well-known topic and that it would take approximately 5–10 minutes to describe (see Table 1). The article was three A4 pages and 2,759 Japanese characters in length. The teacher was separated from the student and instructed to read the article to understand the content. The teacher then summarized the article freely for 5 to 10 minutes. The teacher removed unnecessary content from the summary and then practiced describing the article to the experimenter in his/her own words. At the start of the experiment, the teacher sat face-to-face with the student across a table at a distance of 1.2 meters, with a visual angle of 10.6° for the teacher’s face (see Fig 1B). The temperature of the room was 24.2°C, the illuminance was 913.8 lux (CL-200A, Konica Minolta, Japan) and environmental noise was 34.3 dB (AR814, Smart Sensor, China). The article was placed on a book stand in front of the teacher, who described the article to the student in approximately 5 to 10 minutes in Japanese. The teacher was instructed to speak in a loud and clear voice, and to look the student in the eye while speaking. The student was asked to look the teacher in the eye, to listen carefully to the teacher’s description, and to learn the content. The students were not allowed to ask questions; they were only allowed to use back-channel signals, including head nods and short utterances such as “un,” “hai” and “ee,” which are equivalent to “mmhm,” “uh huh” and “yeah” in English [6, 7, 23–25].

**Table 1:** Results of the face-to face communication condition.

Pair ID	Measurement Period (min:sec.msec)	Density (nods / min)	Mean Phase Difference (ms)	SD (ms)	Kurtosis
1	04:42.85	8.7	110	260	0.6
2	10:01.17	4.7	250	430	-1.0
3	06:00.04	7.3	160	410	-0.6
4	05:31.63	7.1	110	410	-0.3
5	06:48.74	6.2	60	240	1.0
6	06:57.20	21	130	190	4.2
7	07:52.14	14	50	250	3.4
8	06:38.85	8.3	20	200	2.4
9	08:44.31	8.6	100	330	1.3
10	06:04.71	8.6	60	360	0.7
11	05:45.00	9.6	180	410	0.1
12	05:29.77	7.1	80	300	3.6

We instructed the participants to minimize the influence of body movements except head nods, and we imposed the following constraints to help them do so.

- The teacher was not allowed to show the manuscript to the student.
- Neither teacher nor student could change posture significantly.
- Neither teacher nor student could touch the sensor during the experiment.

We used the same procedure in the remote communication condition as in the face-to-face communication condition, except for the following points. The teacher and student sat in separate rooms, and the lecture was given via television. The teacher sat in front of a video camera and described the same article as in the face-to-face communication condition for approximately 5 to 10 minutes. There was no difference in the duration of the teacher’s description between the face-to-face communication and remote communication conditions (t (22) = 0.057, P = 0.955, see Tables 1 and 2). During the practice, we measured the sound level of the teacher’s description every 10 seconds with a digital sound level meter (AR814, Smart Sensor, China), and the volume of the television was adjusted to the actual range of the volume of the teacher’s voice (Mean: 62.3 dB; SD: 5.4 dB). During the experiment, the teacher was asked to look at the camera while speaking, as if speaking face-to-face with the student. The audiovisual information of the teacher was transmitted to a television in the student’s room via a video camera. The student sat in front of the television at a distance of 1.8 meters, and the visual angle of the teacher’s face was 10.6° (see Fig 1C). The student was asked to look the teacher in the eye, to listen carefully to the teacher’s description, and to learn the content. Only back-channel signals were permitted, and the constraints for the experiment were the same as in the face-to-face communication condition.

**Table 2:** Results of the remote communication condition.

Pair ID	Measurement Period (min:sec.msec)	Density (nods / min)	Mean Phase Difference (ms)	SD (ms)	Kurtosis
1	05:29.83	4.5	90	470	-0.5
2	06:40.98	3.6	20	430	-0.1
3	07:19.59	3.0	140	440	-0.8
4	05:27.04	3.3	-50	580	-1.6
5	06:12.68	8.4	290	420	0.9
6	07:02.75	4.4	130	340	1.2
7	06:40.07	3.1	100	430	-0.4
8	05:55.76	6.6	-20	440	1.0
9	06:20.89	2.7	90	550	-1.0
10	06:35.28	6.7	110	310	0.9
11	07:11.30	4.0	-20	350	1.3
12	09:18.63	4.8	60	420	-0.2

### Data Analysis

#### Detection of phase difference

Time-series data on the acceleration of head movements in three axes were recorded with a sampling frequency of 100 Hz. Here, we define a head movement as in a previous study, which is a movement in the vertical (superior and inferior) and longitudinal (anterior and posterior) directions [26]. Thus, we only analyzed the two directions of acceleration shown in Fig 1A. The time-series data of the norm of the accelerations in the vertical and longitudinal directions (*x*, *z*) were calculated as

$$a\left(t_{i}\right)=\sqrt{a_{x}{}^{2}\left(t_{i}\right)+a_{z}{}^{2}\left(t_{i}\right)}\text{ for }i\;=\;0,\;1,\;2,\;\ldots$$ (1)

The interval between *t*_*i*_ and *t*_i+1_ is 10 ms, which is equal to the temporal resolution of the device. As there are differences between individuals in the strength of their nods, *a*(*t*_*i*_) was normalized by

$$a^{\prime }\left(t_{i}\right)=\frac{a\left(t_{i}\right)-\bar{a}}{\sigma_{a}}.$$ (2)

Here, $\bar{a}$ and *σ*_*a*_ are calculated as

$$\bar{a}=\sum_{t_{i}\in T}\frac{a\left(t_{i}\right)}{\left|T\right|},$$ (3)

$$\sigma_{a}=\sqrt{\frac{\sum_{t_{i}\in T}{\left(\bar{a}-a\left(t_{i}\right)\right)}^{2}}{\left|T\right|-1}},$$ (4)

where T represents the total measurement period in each pair. The time-series data *a*′(*t*_*i*_) were smoothed with a moving average of 100 ms to reduce fluctuations due to signal distortion. In a previous study, the durations of posture shifts in head movements were around 400 ms [27]. The moving average of 100 ms means a minimum unit of the same order in the durations of posture shifts in head movements. We calculated the time-series data *a*′(*t*_*i*_) as follows

$$\bar{a^{\prime }}\left(t_{i}\right)=\frac{1}{11}\sum_{l=i}^{i+10}a^{\prime }\left(t_{l}\right)\;\text{for }i\;=\;0,\;1,\;2,\;\ldots$$ (5)

When head nods occurred, the local maximum values, hereafter called peaks, existed in time-series data $\bar{a^{\prime }}\left(t_{i}\right)$. We therefore defined the peak acceleration as the $\bar{a^{\prime }}\left(t_{i}\right)$ that satisfies the following inequality:

$$\bar{a^{\prime }}\left(t_{i}\right)-\bar{a^{\prime }}\left(t_{i\pm 1}\right)>0.$$ (6)

To extract only reliable signals of head nods, we used a threshold amplitude for $\bar{a^{\prime }}\left(t_{i}\right)$ of 2.0 or more. Peaks of 2.0 or more constituted approximately 6% of the total acceleration peaks in all students’ head motions. We used the video data to confirm visually that peaks of 2.0 or more actually corresponded to head nods. Thus, we imposed the following conditions on $\bar{a^{\prime }}\left(t_{i}\right)$:

$$\bar{a^{\prime }}\left(t_{i}\right)\geq 2.0.$$ (7)

After we detected peaks in the acceleration of head nods by student and teacher, we defined the phase difference as the minimum temporal difference (*t*_*j*_-*t*_*i*_) from the time (*t*_*i*_) of a peak in acceleration of the teacher’s head nods to that (*t*_*j*_) of the student. The range of the phase difference was limited to 1.0 s because it has been reported that the maximal delay time for nonverbal synchronization is 1.0 s [12]. Therefore, we imposed the following restriction, in addition to conditions (6) to (7), on the definition of phase difference:

$$-1.0s\leq t_{j}-t_{i}\leq 1.0s.$$ (8)

In the remote communication condition, although the time-series data on the acceleration of teachers’ and students’ head movements were measured in real time, there was a delay in the transfer of the data from the video camera to the television. Although the students were unaware of this delay (they perceived the delayed information and reacted to it as if it were in real time), we needed to measure the delay time and to include it in our calculation of phase difference. To measure the delay time, we transmitted video camera images of a software stopwatch (Online Stopwatch, temporal resolution: 1ms) on a computer screen to the television [28]. We took simultaneous pictures of the time depicted on the stopwatch on the computer screen and the one on the television screen and used the difference between them as the delay time. The mean delay time for 50 trials was approximately 160 ± 13 ms (Mean ± SD). Therefore, the acceleration data for the teacher corresponded to the acceleration data for the student with a time delay of 160 ms in data processing.

#### Analysis of synchronization

Body movement synchronization was defined as the phase difference distribution during the entire communication period. Therefore, the synchronization characteristics are described using statistical analyses of the phase difference distribution of head nodding over the whole measurement period. Specifically, the four statistical measurements are: density, mean phase difference, standard deviation (SD) and kurtosis. First, we introduced the density of the frequency of phase difference, defined as the frequency per minute within each pair. Density is an indicator of synchronization activity. Second, we introduced the mean phase difference, defined as the mean of the distribution. The mean phase difference is an indicator of the synchronization direction, that is, whether the speaker or the listener leads the body movements in the synchronization built during communication. Third, we introduced the SD, defined as the spread of the phase difference distribution. Fourth, we introduced kurtosis, defined as the degree of convergence to the mean phase difference in the distribution. The SD and kurtosis are indicators of the synchronization strength.

## Results

**Fig 2:**
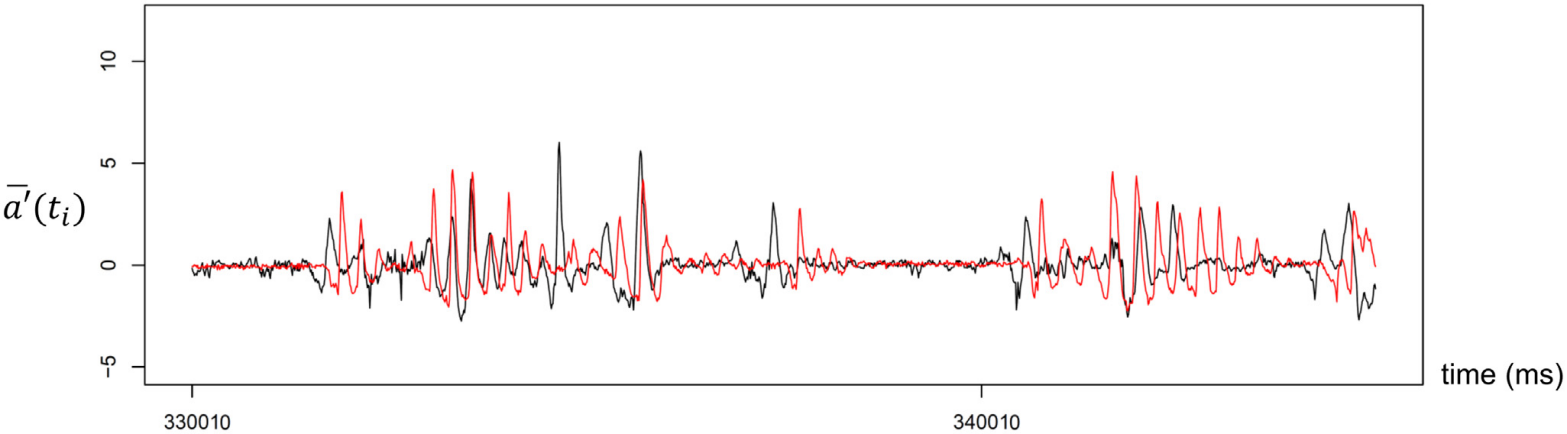
Typical time series data for head nods in the face-to-face communication condition. The black line indicates the teacher’s acceleration data, and the red line shows the student’s acceleration data.

**Fig 3:**
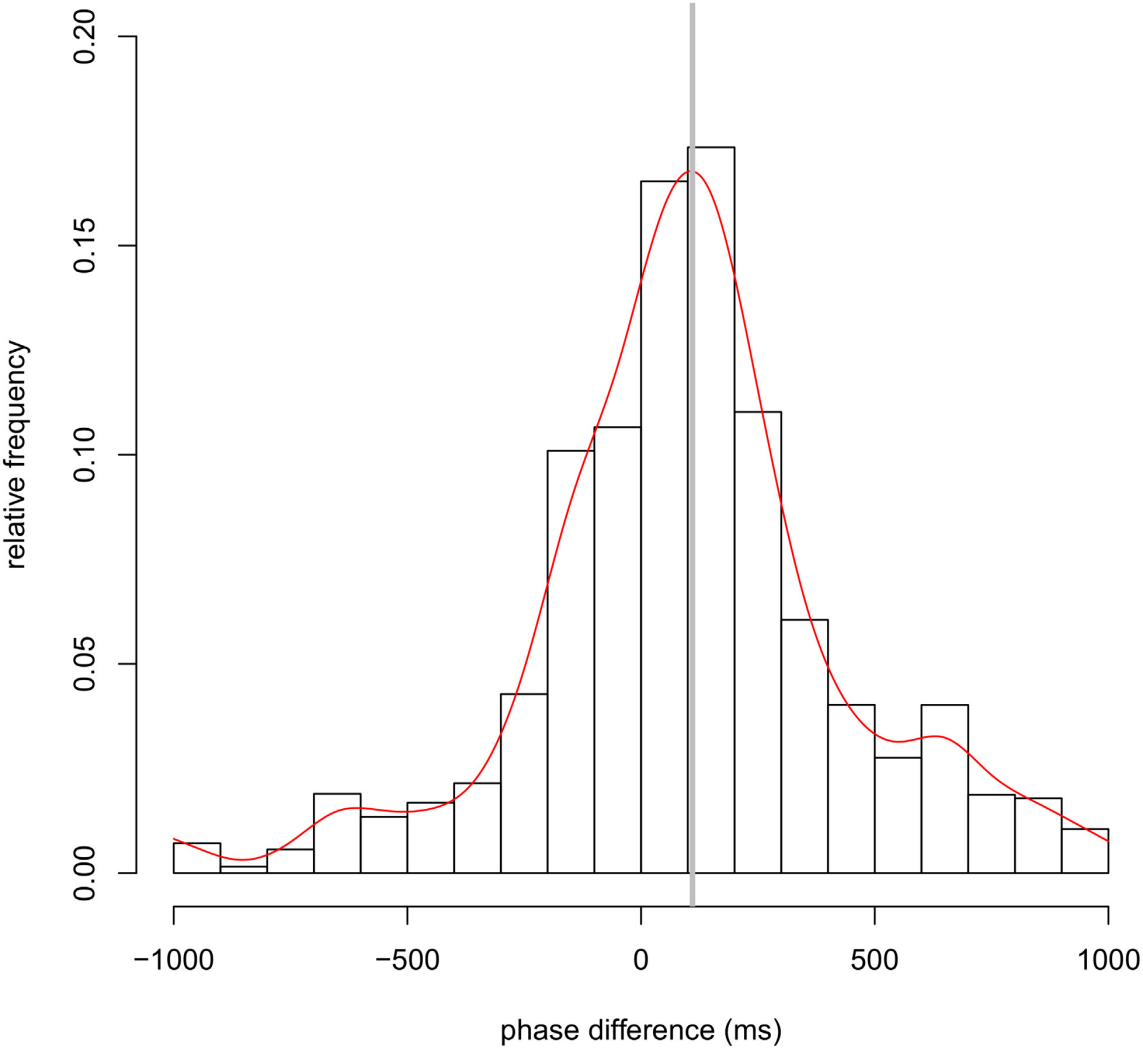
Total results from the face-to-face communication condition. Distribution of the mean relative frequency of synchronized head nods across all pairs in the face-to-face communication condition. A smoothing spline curve (red line) is fitted to the mean relative frequency of synchronized head nods across all pairs and the vertical gray line shows the mean phase difference in face-to-face communication. The horizontal axis represents the phase difference when head nod synchronization occurred, and the vertical axis indicates the frequency of head nod synchronization. Negative values on the horizontal axis indicate that the student’s head nod occurred before that of the teacher, whereas positive values indicate the reverse.

We detected the synchronization of head nods for each pair of participants using the phase difference distribution. Fig 2 illustrates typical time series data for head nods in the face-to-face communication condition, and we plotted the relative distribution of the phase difference of all student–teacher pairs. Fig 3 shows the total results from the face-to-face communication condition (also see S1 Table). Total results are obtained by the overall means of the relative frequency of head nods in each class (intervals of 100 ms) across all pairs. In Fig 3, the horizontal axis represents the phase difference, and the vertical axis indicates the relative frequency of head nods. Negative values on the horizontal axis indicate that the student’s head nod occurred before the teacher’s, whereas positive values indicate the reverse.

Table 1 shows the results for each pair in the face-to-face communication condition (also see S1 Fig). In the face-to-face communication condition, as shown in Fig 3, the distribution of phase difference in head nods was symmetric and converged on the vicinity of the center. In the face-to-face communication condition, the mean density across pairs was 9.2 nods/min (SD = 4.2 nods/min). The overall mean (across pairs) of the mean phase differences was 110 ms, and the mean of the SDs across pairs was 320 ms. The mean kurtosis across pairs was 1.3 (SD = 1.7).

**Fig 4:**
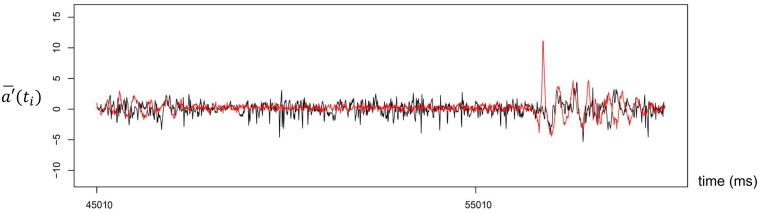
Typical time series data on head nods in the remote communication condition. The black line indicates the teacher’s acceleration data, and the red line shows the student’s acceleration data.

**Fig 5:**
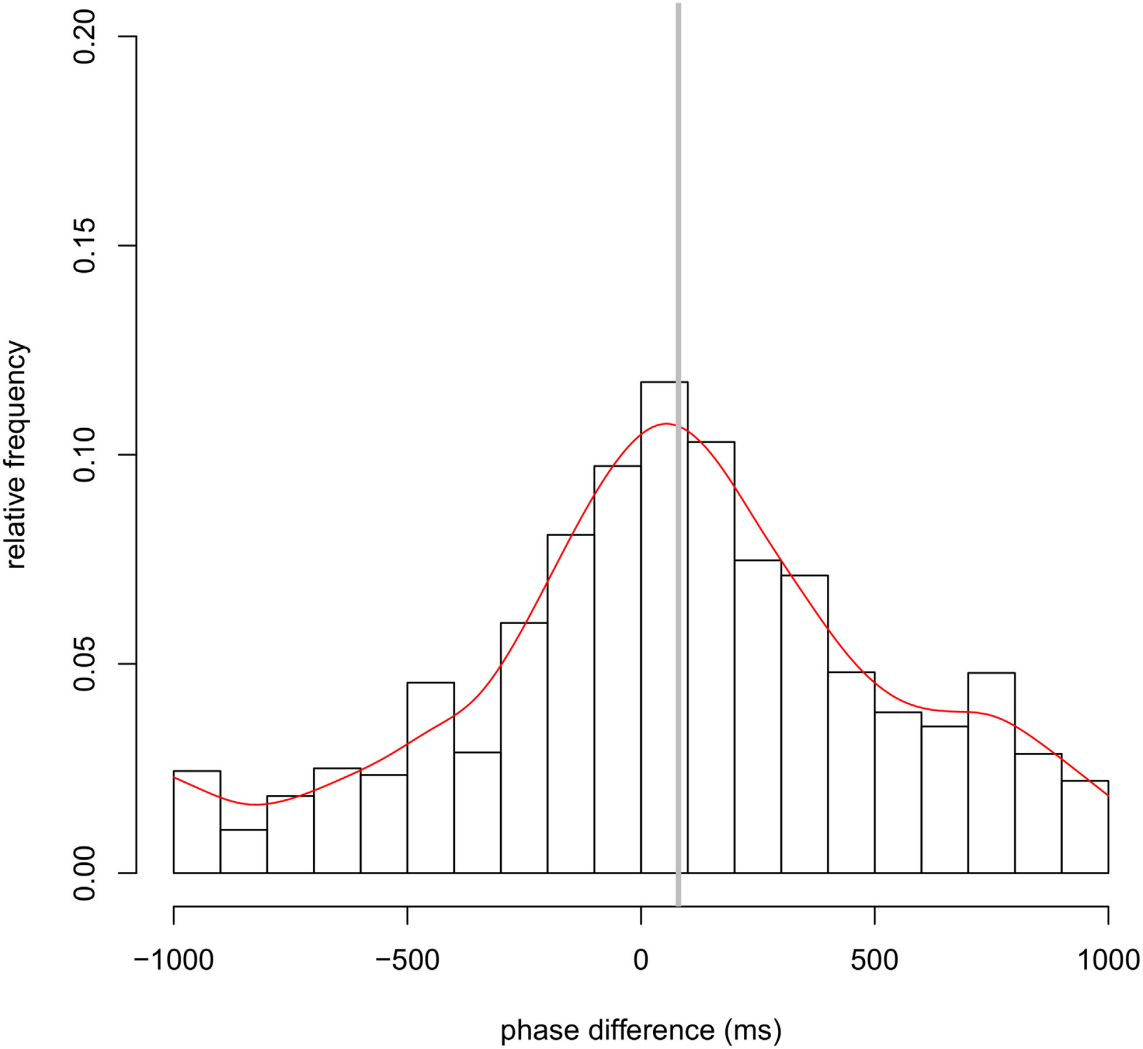
Total results from the remote communication condition. Distribution of the mean relative frequency of synchronized head nods across all pairs in the remote communication condition. A smoothing spline curve (red line) is fitted to the mean relative frequency of synchronized head nods across all pairs and the vertical gray line shows the mean phase difference in remote communication. The horizontal axis represents the phase difference when head nod synchronization occurs, and the vertical axis indicates the frequency of head nod synchronization. Negative values on the horizontal axis indicate that the student’s head nod precedes that of the teacher, whereas positive values indicate the reverse.

Fig 4 illustrates typical time series data for head nods in the remote communication condition. Table 2 shows the results for each pair in the remote communication condition (see also S2 Fig) and Fig 5 shows the total results from the remote communication condition (see also S1 Table). In the remote communication condition, the distribution of phase difference in head nods converged on the vicinity of the center. The mean density was 4.6 nods/min (SD = 1.7 nods/min). The overall mean (across pairs) of the mean phase differences was 80 ms, and the mean of the SDs across pairs was 430 ms. The mean kurtosis across pairs was 0.1 (SD = 0.9).

**Fig 6:**
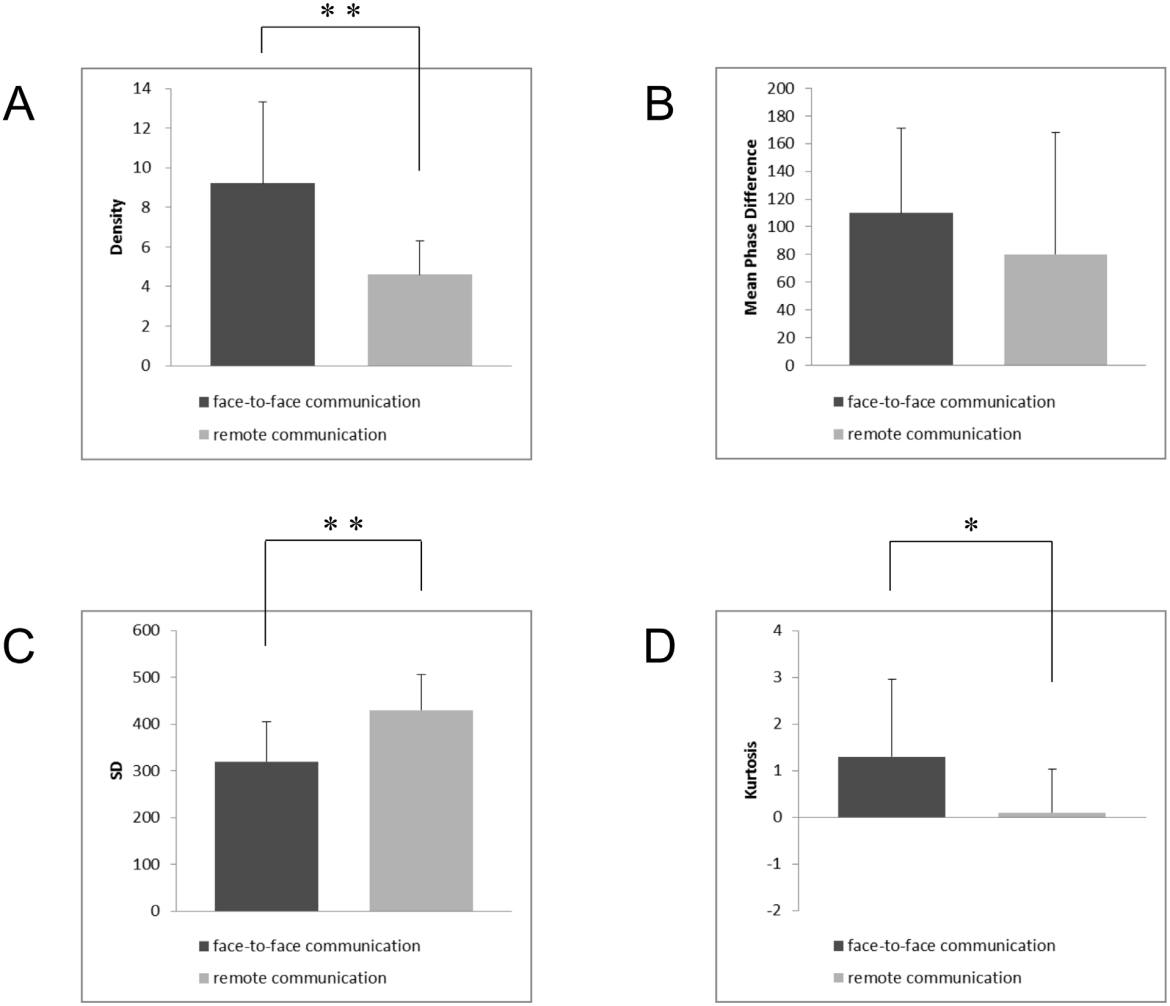
Comparison of results between the face-to-face communication and remote communication conditions. (A) shows the mean density in the face-to-face communication and remote communication conditions. The error bars represent the standard deviations of the means. (B) shows the mean phase difference in the face-to-face communication and remote communication conditions. The error bars represent the standard deviations of the means. (C) shows the mean SD in the distribution of the phase differences in the face-to-face communication and remote communication conditions. The error bars represent the standard deviations of the means. (D) shows the mean kurtosis in the distribution of the phase differences in the face-to-face communication and remote communication conditions. The error bars represent the standard deviations of the means, *: *p* < .05, **: *p* < .01, unpaired t-test.

Unpaired t-tests indicated that the densities in the face-to-face communication condition was significantly higher than those in the remote communication condition (t(22) = 3.420, P = 0.002; see Fig 6A, but the mean phase differences did not show a significant difference between the conditions (t(22) = 0.937, P = 0.359, see Fig 6B. Unpaired t-tests also revealed that SDs in the face-to-face communication condition were significantly smaller than those in the remote communication condition (t(22) = –3.405, P = 0.003; see Fig 6C and kurtoses in the face-to-face communication condition were significantly higher than those in remote communication condition (t(22) = 2.098, P = 0.048; see Fig 6D.

## Discussion

In this study, we defined the phase difference of head nods during the entire communication period and the characteristics of head nod synchronization as the phase difference distribution. Specifically, the phase difference distribution was characterized using four statistical measurements: the density, the mean phase difference, the SD, and the kurtosis. To verify the validity of our definition, we applied this method to two human communication situations focusing on the influence of visual interaction in the synchronization of head nods: the face-to-face communication condition with visual interaction and the remote communication condition with unidirectional visual perception. As a result, the difference between the phase difference distributions in these communication situations was clearly shown. Although the mean phase differences in head nods did not differ significantly between the face-to-face communication and remote communication conditions, there were significant differences in the densities, the SDs and kurtoses in the phase difference distributions of head nod synchronization between the face-to-face communication and remote communication conditions. We can use these results to clarify the characteristics of body movement synchronization through the features of the phase difference distribution.

First, the density, the SD and the kurtosis of the phase difference distribution differed between the face-to-face communication condition with visual interaction and the remote communication condition with unidirectional visual perception. Thus, visual interaction in the face-to-face communication condition led to a large density of synchronized head nods and a small spread (small SD and large kurtosis) compared with the remote communication condition. This means that visual interaction resulted in higher synchronization activity and strength. Most importantly, these differences clearly showed the mechanism of body movement synchronization in human communication. Schmidt et al. (1990) reported that visually coupled perceptions between individuals is important for the coordination of body movements [20]. In both communication conditions in the present study, the teachers presented the same auditory information to the students. However, these conditions differed in their visual modality, because the teachers could see the students’ back-channel signals in the face-to-face communication condition, but this information was not available to the teachers in the remote communication condition. This interaction through the visual channel may contribute to mutual entrainment in nonverbal synchronization, because synchronization phenomena are established by the mutual entrainment mechanism based on interaction between nonlinear oscillators from a theoretical viewpoint [18, 19]. This finding will play an important role in the elucidation of the mechanism of nonverbal synchronization in face-to-face communication and the application of remote communication technologies.

Second, there was no difference in the mean phase difference between face-to-face communication with visual interaction and remote communication with unidirectional visual perception. This means that visual interaction in the head nod synchronization did not affect the mean phase difference. The mean phase difference is an indicator of the synchronization direction, that is, whether the speaker or listener leads the body movements in the synchronization built during communication. The speaker’s head nods tended to slightly lead the listener’s head nods in both communication conditions. In recent years, the mechanism of nodding in face-to-face communication has been reported. Bavelas et al. (2002) reported that the nodding of the listener occurred in a gaze window, which is a temporal window of mutual gaze created by the speaker looking towards the listener [29]. In addition, according to Stivers (2008), nods by a listener act as a sign of alignment with the activity of speaking and affiliation through a claim of access to the speaker’s stance, either indirectly or directly [30]. These studies well represent the mechanism of the occurrence and function of nodding. However, in this study, the synchronization characteristic of head nods was detected even in the remote communication condition without mutual gaze related to visual interaction, in which there was no difference in the mean phase differences between the face-to-face and remote communication conditions. This therefore shows the synchronization direction of head nods may be attributed to the listener’s alignment, that is, the listener’s adaptive behavior to the speaker’s multimodal behavior, even in remote communication.

As discussed above, head nod synchronization could be characterized by the phase difference distribution. It is possible that body movement synchronization is achieved by simultaneity perception. In previous studies, simultaneity perception has been studied through the distribution of simultaneity judgment. In the field of cognitive psychology, the point of subjective simultaneity (PSS) is commonly used, which is an indicator of subjective simultaneity in sensory processing by a human perceptual system. The PSS is obtained by the mean of a distribution of simultaneous responses, and it has been reported that the PSS differs from physical simultaneity in multisensory integration [31–38]. Interestingly, the present study indicates that the mean phase difference in nonverbal synchronization has the same tendency as the PSS, as the mean phase difference (corresponding to the stimulus onset asynchrony at the PSS) was not zero (i.e., physically perfect synchronization). Another indicator is the temporal window of integration, which means the width of simultaneous perception [31–33, 38–41]. The temporal window of integration is calculated as the standard deviation (SD) of a distribution of simultaneous responses. In this study, the mean phase difference was 110 ms and SD was 320 ms in the face-to-face communication condition. In particular, Figs 3 and 5 show that the phase difference distribution changes in shape for every 100 ms (see also S3 and S4 Figs). From this perspective, in the future we need to investigate the point of subjective synchronization during communication and the temporal window of synchronization as the effective width of synchronization.

In this study, we applied our definition to human communication in which the roles of speaker and listener were defined. However, in the future it will be necessary to examine other factors such as mutual talk to clarify the influence of other interactions as a cause of synchronization in human communication. In addition, there is a need to examine the verbal factor in which the listener can only hear the speaker but has no visual access to the speaker in order to determine the unique influence of verbal and nonverbal behavior. Also, our data were obtained only from Japanese conversations and head nods. Therefore, there is a further need to examine the influence of other languages, different cultures and nonverbal signals. Although remote communication has been developed to approximate face-to-face communication, remote communication remains inadequate compared with face-to-face communication [42, 43]. Therefore, our findings will prompt research on future communication technology based on nonverbal synchronization in face-to-face and remote communications. We believe that these findings are useful in detecting nonverbal synchronization in various human communication situations.

## Supporting Information

S1 Fig Distribution of the relative frequency of synchronized head nods for each pair in the face-to-face communication condition. (TIF)

S2 Fig Distribution of the relative frequency of synchronized head nods for each pair in the remote communication condition. (TIF)

S3 Fig Distribution of the mean relative frequency of synchronized head nods within a range of 0 to 100 ms across all pairs in face-to-face communication. Red lines show smoothing spline curves.
(TIF)

S4 Fig Distribution of the relative frequency of synchronized head nods within a range of 0 to 100 ms for each pair in face-to-face communication. Red lines show smoothing spline curves.
(TIF)

S1 Table Mean relative frequency of synchronized head nods for every 100 ms across all pairs (in the face-to-face communication and remote communication conditions). (DOCX)
