# Supplementary material for: Detection of Nonverbal Synchronization through Phase Difference in Human Communication
Source: PLoS One. 2015 Jul 24;10(7):e0133881. doi: 10.1371/journal.pone.0133881 (PMC4514884; doi:10.1371/journal.pone.0133881)
Supplement: S1 Table — (DOCX) [file pone.0133881.s005.docx]

| Table S1. Mean relative frequency of synchronized head nods for every 100 ms across all pairs (in the face-to-face communication and remote communication conditions) | | |
| --- | --- | --- |
|  | | |
| Temporal Range | Face-to-face Communication Condition | **Remote Communication Condition** |
| ― 900 ~ ― 1000 | 0.025 | 0.024 |
| ― 800 ~ ― 900 | 0.011 | 0.010 |
| ― 700 ~ ― 800 | 0.014 | 0.018 |
| ― 600 ~ ― 700 | 0.019 | 0.025 |
| ― 500 ~ ― 600 | 0.019 | 0.023 |
| ― 400 ~ ― 500 | 0.041 | 0.046 |
| ― 300 ~ ― 400 | 0.030 | 0.029 |
| ― 200 ~ ― 300 | 0.054 | 0.060 |
| ― 100 ~ ― 200 | 0.082 | 0.081 |
| 0 ~ ― 100 | 0.098 | 0.097 |
| 0 ~ 100 | 0.128 | 0.117 |
| 100 ~ 200 | 0.104 | 0.103 |
| 200 ~ 300 | 0.082 | 0.075 |
| 300 ~ 400 | 0.079 | 0.071 |
| 400 ~ 500 | 0.044 | 0.048 |
| 500 ~ 600 | 0.044 | 0.038 |
| 600 ~ 700 | 0.033 | 0.035 |
| 700 ~ 800 | 0.046 | 0.048 |
| 800 ~ 900 | 0.030 | 0.029 |
| 900 ~ 1000 | 0.019 | 0.022 |
